# Filling Teeth

**Published:** 1846-09

**Authors:** 


*Filling Teeth.*—A great deal of labor, mechanical tact, and experience are required to fill a tooth so as to secure its preservation. Few dentists perform this operation in a proper and thorough manner, and hence it is that it so frequently fails to accomplish the object intended to be secured by it. Although by far the most important, and at the same time the most difficult operation in dental surgery, it is performed by a large majority of practitioners in an exceedingly imperfect and bungling manner, and yet we cannot believe that all who fill teeth thus, are incapable of doing it better. We believe that many could perform the operation in a neater and more substantial manner, if they would bestow upon it six or eight times the amount of labor which they are accustomed to do. It is impossible for any one to fill properly fifteen or thirty, or forty cavities in teeth, a day, as many do. As a general rule, from an hour to an hour and a quarter constant, hard labor, is required to prepare a cavity in a tooth and fill it in a neat and durable manner. We often find it necessary to labor constantly three, and sometimes five hours upon a single filling; and it rarely happens that we can accomplish the operation to our own satisfaction in less than one hour.

An individual, calling himself a dentist, visited us a short time since, and in speaking of this operation remarked, that he frequently put in fifty fillings a day, and it is no uncommon thing to hear dentists speak of filling from twenty-five to thirty teeth a day. Now, such practitioners must be either ignorant of the manner of performing the operation or dishonest, and in either case ought not to be entrusted with the treatment of the diseases of organs so valuable as the human teeth; yet, these very men are often liberally patronised, and simply for the reason that they propose to operate for low prices, and promise, in their advertisements, to make their operations superior to those of other dentists. But even among practitioners of a much higher grade, the operation in question, is not performed with the care its importance demands, and our object in calling attention to it at this time, is to notice the error with regard to the length of time required to insert a good filling, into which many seem to have fallen. We may at another time take occasion to speak of the manner of preparing the cavity, introducing the gold, and finishing the surface of the filling.—*Balt. Ed.*

